# The Evolution of Extended Platelet-Rich Fibrin Membranes for Socket Grafting: Part One: Technical Development of Protocols

**DOI:** 10.3390/dj13120604

**Published:** 2025-12-16

**Authors:** Nathan E. Estrin, Alan Rene Espinoza, Paras Ahmad, Jean-Claude Imber, Nima Farshidfar, Richard J. Miron

**Affiliations:** 1 College of Dentistry and Dental Clinics, The University of Iowa, Iowa City, IA 52242, USA; drnathan@estrinperiodontics.com; 2 Department of Research, Advanced PRF Education, Jupiter, FL 33477, USA; 3 Private Practice, Global Dental Baja, Mexicali 21200, Mexico; 4 Department of Periodontology, University of Bern, 3010 Bern, Switzerland; jean-claude.imber@unibe.ch (J.-C.I.);

**Keywords:** alveolar ridge augmentation, bone substitute, dermal fillers

## Abstract

**Background:** Platelet-rich fibrin (PRF) is an autologous biomaterial utilized as an adjunct in dental implant surgeries owing to its significant biocompatibility, supra-physiological concentration of growth factors, and ability to speed either soft or hard tissue regeneration. **Methods:** Today, PRF is available in both solid and liquid forms with an average resorption period of roughly 2 weeks. While various research endeavors have attempted to utilize Solid-PRF as a barrier membrane in guided bone regeneration (GBR) and various other applications, its two-week resorption period has limited its use as a solo “barrier” membrane owing to its faster-than-ideal resorption properties. **Results:** Recent studies have demonstrated that by heating and denaturing Liquid-PRF/albumin, the resorption properties of the heated albumin gel could be extended from 2 weeks to 4–6 months by utilizing the Bio-Heat technology. This emerging technology was given the working name ‘extended-PRF’ or e-PRF, with many clinical indications being proposed for further study. Numerous clinicians have now utilized extended-PRF (e-PRF) membranes as a substitute for collagen barrier membranes in various clinical applications, such as guided tissue/bone regeneration, recession coverage, and lateral window sinus lifts. **Conclusions:** This two-part case series paper aims to first illustrate the evolution of techniques developed taking advantage of this new technology in clinical practice for alveolar ridge preservation. This includes four different methods of fabrication of e-PRF along with its application in clinical practice. This article discusses the clinical outcomes, including the advantages/disadvantages of utilizing each of the four separate techniques to prepare and utilize e-PRF membranes for ridge preservation.

## 1. Introduction

Autologous regenerative biomaterials such as platelet-rich fibrin (PRF) are being more frequently utilized owing to their ability to speed both soft and hard tissue regeneration while also possessing antibacterial properties [1,2,3,4,5,6,7]. Furthermore, much additional research has investigated improvements in centrifugation protocols, tube quality, and clinical applications [8,9,10,11,12,13,14]. Marx et al. [15] were the first to postulate that autologous growth factors could be concentrated by collecting the patient’s own blood, which could then be centrifuged and eventually administered locally to facilitate tissue regeneration.

Initially, anticoagulants were included in the collection tubes of the ‘first’ generation of autologous platelet concentrates, termed platelet-rich plasma (PRP), to prevent blood clotting, which would allow plenty of time for centrifugation to be carried out over a 30 min processing technique [16]. Nevertheless, it was later discovered by clinical scientists that blood clotting is one of the first and most pivotal steps to wound healing; therefore, anticoagulant removal was advised [17,18]. Since PRF excludes the use of anticoagulants, a shorter centrifugation protocol was utilized, leading to a faster protocol that is more easily implemented in clinical practice [19]. To maximize the size of the fibrin clot generated, clinicians are recommended to transfer the tubes filled with blood to the centrifugation device within 90 s from the time the blood is first drawn [20].

The first step towards successful dental implant therapy is managing the dimensional alterations of both hard and soft tissues following dental extractions [21]. In order to reduce post-extraction dimensional changes, ridge preservation has been accomplished utilizing a combination of bone grafts, membranes, and growth factors [22]. PRF has been utilized during ridge preservation procedures due to its regenerative potential, including its ability to promote faster revascularization of the defect areas and recruit surrounding progenitor cells for healing [23]. Most commonly, both solid and liquid forms of PRF are combined with bone particulate to create “sticky bone,” which, in addition to adding growth factors to the bony scaffold, has the added advantage of easier handling [24,25]. Additionally, Solid-PRF has been utilized by clinicians as a solo membrane in ridge preservation; however, due to its quick two-week resorption period, it is not considered an ideal biomaterial over traditional barriers, including collagen membranes or PTFE membranes [26]. This is because if the membrane resorbs prior to complete wound closure, the grafted socket is left vulnerable with potential for bone graft particle loss from the defect.

Interestingly, a number of recent studies have demonstrated that PRF could be significantly extended from its typical 2–3-week resorption period to greater than 4 months (extended-PRF; e-PRF) by heating a liquid platelet-poor plasma (PPP) layer (denaturing albumin) using the Bio-Heat technology [26,27]. The heated version of plasma, which has now been utilized in many areas of medicine and dentistry [28], has recently been the basis of intensive pre-clinical and clinical research owing to its extended working properties. Since one of the main limitations of PRP/PRF has historically been its short in vivo turnover rate, these extended-PRF membranes can be used as substitutes for collagen membranes in guided bone regeneration (GBR) procedures requiring a typical ‘barrier’ function that protects bone regeneration from faster-growing soft tissues [28].

A split-mouth study by Abdulhak et al. [29] compared albumin-PRF (alb-PRF) to a connective tissue graft for recession coverage, demonstrating superior, thicker gingiva in the alb-PRF group but less increase in keratinized tissue width. Additionally, our group reported that this novel 100% autologous membrane can be utilized safely and effectively as a membrane for closure in lateral window sinus lifts [30]. This article is part one of a two-part study, comprising a technical description and subsequent comparative evaluation of the four different iterations of e-PRF that can be fabricated/utilized in alveolar ridge preservation and discusses the evolution of this emerging technique.

## 2. Study Design

Institutional Review Board (IRB) approval was obtained from Sterling IRB (ID: 13180-NEstrin). The investigations were conducted in accordance with the principles outlined in the Declaration of Helsinki. All patients were treated in a private practice setting (Lakewood Ranch Dental, Sarasota, FL, USA), and informed consent was obtained for all patients. This current paper is part one of a two-part series structured as a narrative technical case series describing the evolution of e-PRF membranes in ridge preservation, with part two as a comparative study of these novel techniques to traditional collagen membranes. The purpose of this first article is to provide, in detail, the technical differences between the different iterations of utilizing e-PRF in ridge preservation with case examples and clinical observations discussed. Formal comparisons to conventional barrier membranes will occur in the second part of this series.

## 3. Fabrication of e-PRF Membranes

**Figure 1:**
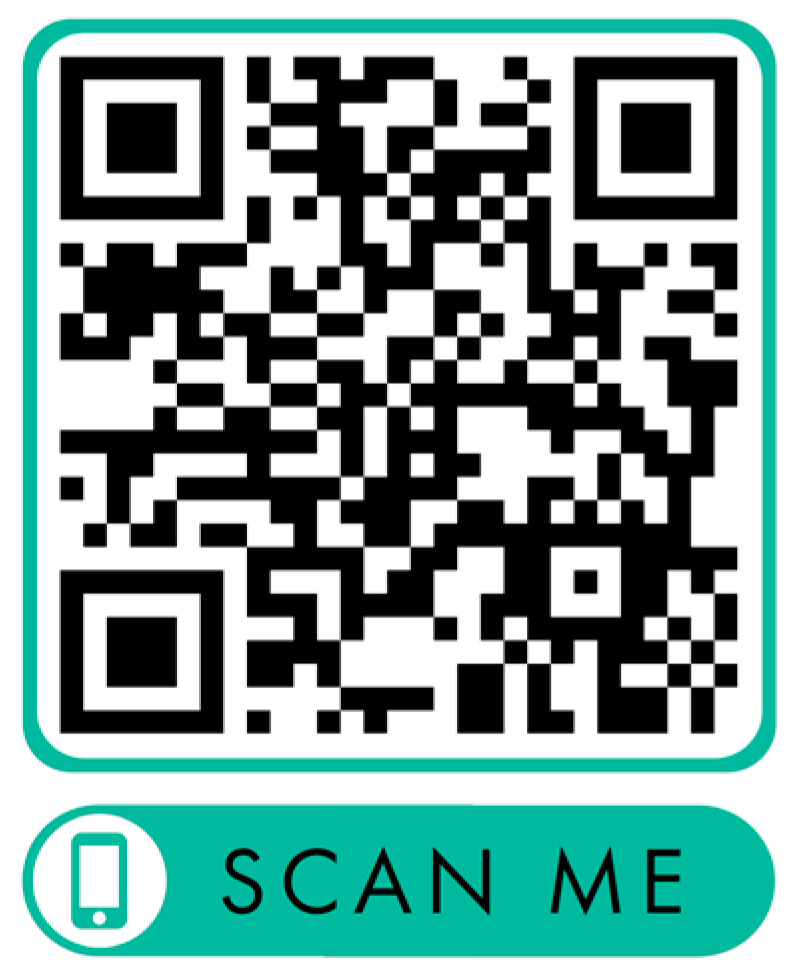
QR Code 1. How to make an e-PRF membrane.

The initial fabrication of an e-PRF membrane followed the initially proposed standard protocol according to those previously published [26,27]. Briefly, an 18 G needle is utilized to draw two 10 mL Liquid-PRF tubes of blood from the patient and centrifuged at 700× *g* for 8 min utilizing a horizontal centrifuge (Bio-PRF, Jupiter, FL, USA). After centrifugation, one syringe is utilized to draw 2–4 mL of the platelet-poor plasma (PPP) layer from one blue tube and is placed in the Bio-Heat Device (Bio-PRF, Jupiter, FL, USA) for 10 min at a temperature of 75 degrees Celsius for serum albumin denaturation to produce the plasma gel. A second syringe is then utilized to draw 2–4 mL of Liquid-PRF from the buffy coat zone. The denatured albumin gel is then placed and condensed in a custom Bio-Heat tray (Bio-PRF, Jupiter, FL, USA) to the desired shape. The Liquid-PRF from the buffy coat is then removed and utilized to saturate the albumen gel in the Bio-Heat tray (Bio-PRF, Jupiter, FL, USA) reintroducing growth factors and clotting factors within the final e-PRF membrane composition. The gel and Liquid-PRF were then left for 10–15 min to set prior to use (Figure 1, QR Code 1).

**Figure 2:**
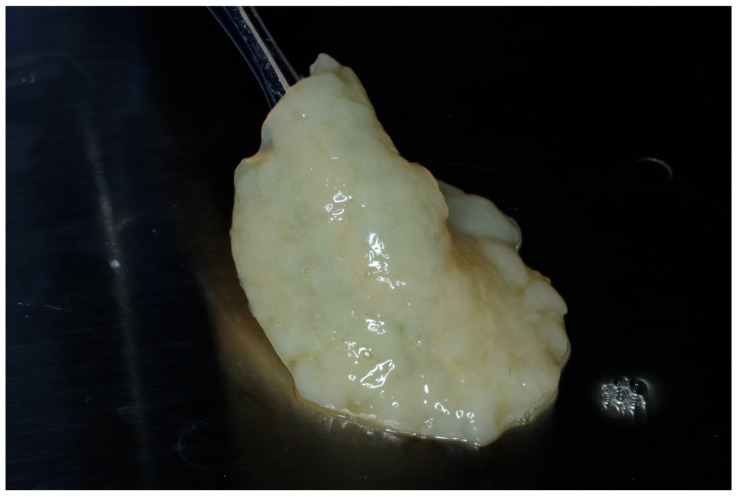
Final fabrication of an e-PRF membrane to be utilized for ridge preservation.

Alternatively, the albumin gel can be pre-mixed with the buffy coat layer of the unheated Liquid-PRF tube, utilizing a female–female luer lock before being deposited in the tray in the desired shape. For this method, the albumin gel is placed in the Bio-Cool (Bio-PRF, Jupiter, FL, USA) at a temperature of 8 degrees Celsius for 2 min prior to mixing with the Liquid-PRF. The final e-PRF membrane is depicted in Figure 2.

### 3.1. Iteration #1—Use of e-PRF as a Sole Barrier Membrane

**Figure 3:**
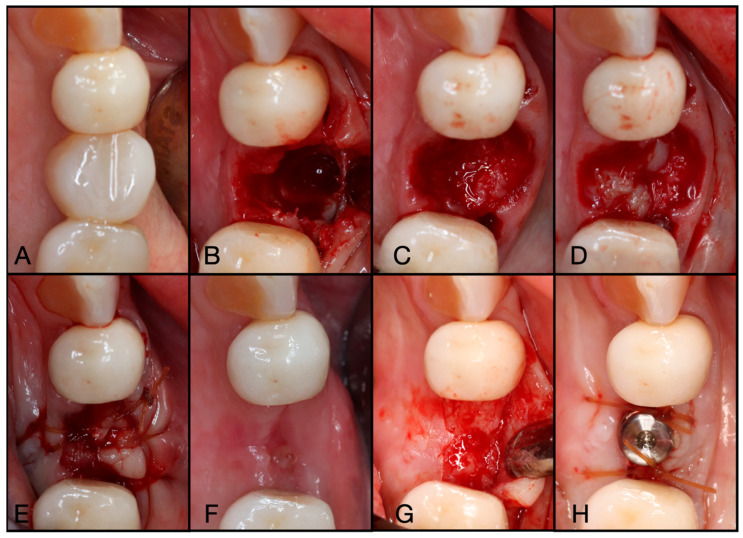
(**A**) Pre-operative clinical photograph. (**B**) After extraction of tooth #44 (due to recurrent decay), the socket was degranulated with a spoon excavator, leaving a four-wall socket with a thin buccal plate. (**C**) Both Solid- and Liquid-PRF were utilized with particulate bone allograft to create “Sticky bone,” which was then moderately packed in the socket. (**D**) The e-PRF membrane was then placed over the grafted socket and (**E**) sutured with 4–0 chromic gut in mattress fashion. (**F**) The patient was brought back for a two-week post-operative evaluation. Note the excellent healing and complete wound closure. (**G**) Upon implant placement, notice the contour of the ridge was well maintained with excellent bone quality. (**H**) A 3.4 × 10.5 mm BioHorizons Tapered Pro implant was placed. Due to a sufficient torque value, a healing abutment was placed, and final suturing was completed with 4-0 chromic gut in a single interrupted fashion.

**Figure 4:**
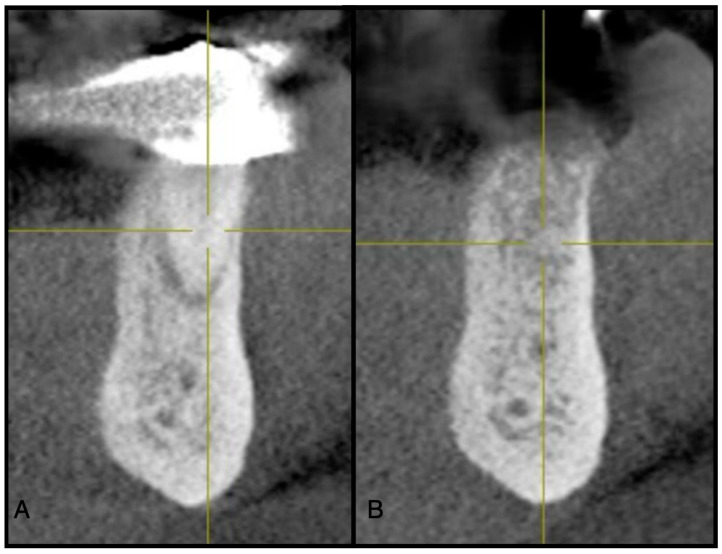
(**A**) Pre-operative CBCT at site #44. (**B**) Three-month post-operative radiograph of site #44 showing adequate ridge dimensions for implant placement after utilizing an e-PRF membrane over bone allograft particulate.

**Figure 5:**
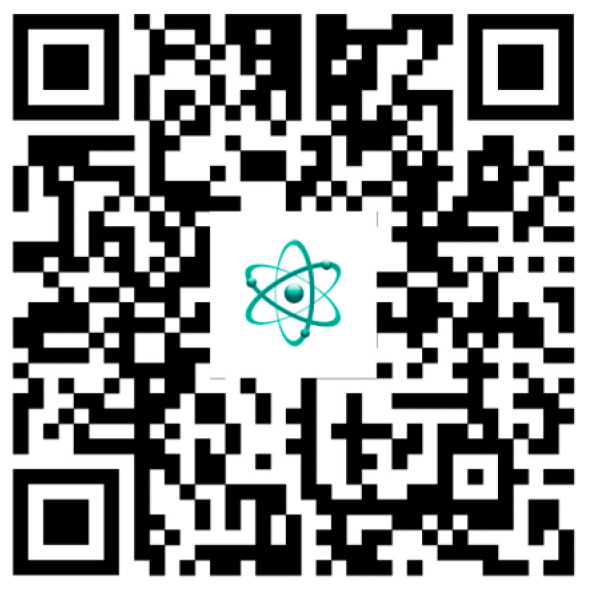
QR Code 2. Utilizing an e-PRF membrane for ridge preservation.

The first use of e-PRF membranes for extraction site management was utilized as a sole barrier membrane over top of a bone allograft, as presented in the clinical case, Figure 3 and Figure 4. The e-PRF membrane was utilized as an unaccompanied membrane in the ridge preservation of tooth #44, successfully leading to implant placement in a healthy 67-year-old female. An accompanying video of a different case example is presented in Figure 5 QR Code 2.

**Figure 6:**
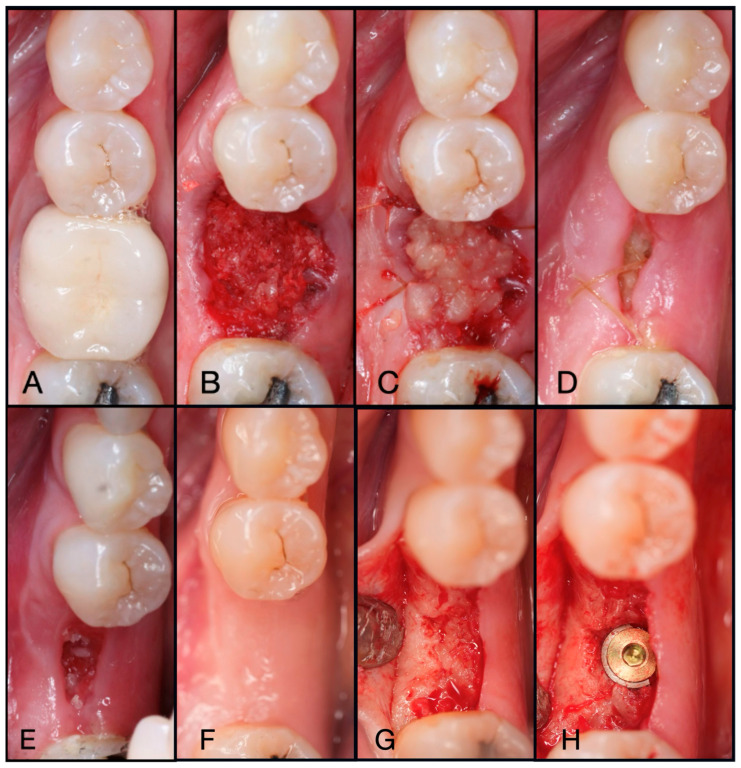
(**A**) Pre-operative clinical photograph of tooth #36, which presented with recurrent decay underneath the existing crown. (**B**) Following atraumatic extraction of tooth #36, the socket was degranulated with a spoon excavator. Then, “Sticky bone” was packed within the socket utilizing a mixture of both Solid-PRF and Liquid-PRF with particulate bone allograft. (**C**) The e-PRF membrane was then placed over the grafted socket and sutured with 4-0 chromic gut in mattress fashion. (**D**) The patient was seen at 8 days post-operatively with noted accelerated healing with nearly complete wound closure. (**E**) At the two-week post-operative evaluation, the e-PRF membrane was now partially gone, with bone particulate exposed and even embedded within the surrounding soft tissue. (**F**) Despite partial loss of the e-PRF membrane, soft tissue healed uneventfully. (**G**) Upon re-entry, adequate ridge width was observed and (**H**) implant placement followed with successful outcomes.

Much success was observed by utilizing e-PRF membranes alone in this fashion. Specifically, the dimensional width of the ridge is maintained with complete soft tissue closure achieved prior to resorption of the e-PRF membrane and bone graft without minimizing the width of keratinized tissue. However, it was observed that in a few cases, the fragility of the e-PRF membrane (especially in those aggressively brushing over the area or eating solid foods post-surgery) led to potential damage and/or loss of the e-PRF membrane. This was one of the main reported observations of the technique, though in no case was the graft material lost or the ridge preservation unsuccessful. Figure 6 demonstrates that despite observing quite prolific healing 8 days post-operatively by utilizing the e-PRF membrane alone, it was further reported that while the e-PRF membrane was lost at the two-week mark due to poor patient compliance, leaving the bone particulate vulnerable to the oral cavity, upon re-entry, the ridge width was sustained and an implant was placed (Figure 6).

### 3.2. Layering a Solid-PRF Membrane over Top of the e-PRF Membrane

**Figure 7:**
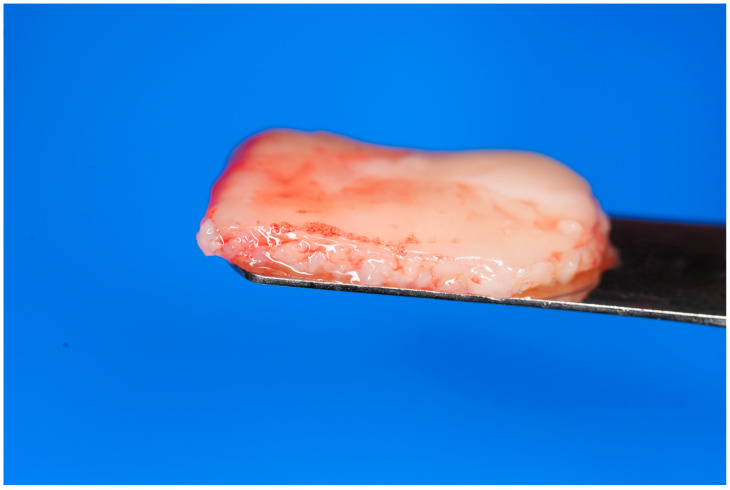
Layering of a Solid-PRF membrane over e-PRF. During the processing of a standard e-PRF membrane, if the Solid-PRF is added right after mixing the Liquid-PRF with the albumin gel (before setting), the Solid-PRF membrane will be incorporated and clotted within the e-PRF membrane (dual-layered technique). By incorporating the layers together, the clinical handling thereafter is much easier/superior.

**Figure 8:**
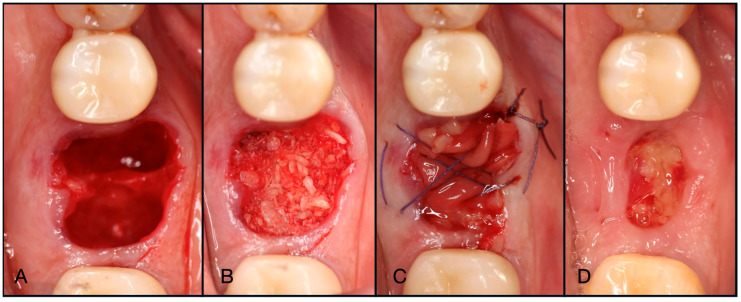
(**A**) Atraumatic extraction of #46, which was extracted due to a failed endodontic procedure. (**B**) After thorough degranulation, the defect was packed with “Sticky bone” created by mixing both Solid- and Liquid-PRF with particulate bone allograft. (**C**) A dual-layered membrane with Solid-PRF overtop an e-PRF membrane was placed to protect the barrier membrane and sutured with 4-0 Vicryl sutures in mattress fashion. (**D**) A two-week post-operative evaluation demonstrates complete resorption of the Solid-PRF, leaving the e-PRF membrane in place to continue to function as the barrier membrane in this case.

**Figure 9:**
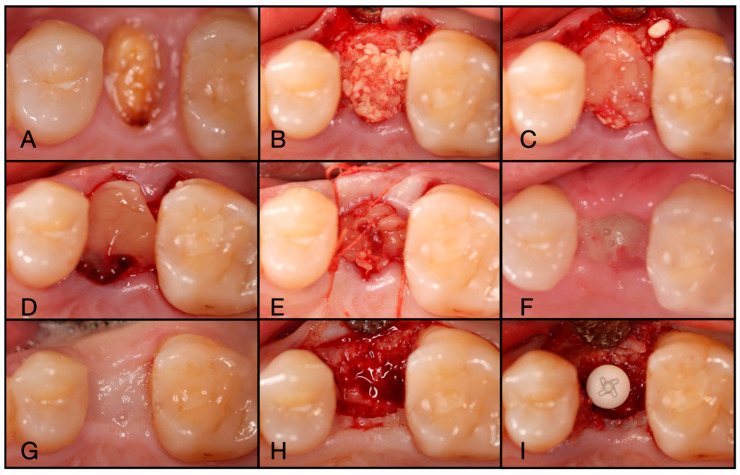
(**A**) Pre-operative clinical photograph of tooth #25, which presented a fractured crown to the gingival margin. (**B**) After atraumatic extraction of tooth #25, the socket was degranulated with a spoon excavator and packed with “Sticky bone” created by mixing both Solid- and Liquid-PRF with particulate bone allograft. (**C**) An e-PRF membrane was placed, followed by a (**D**) Solid-PRF membrane overtop. (**E**) 4-0 Vicryl sutures were utilized in a criss-cross fashion to secure the dual-layered membrane in place. (**F**) The patient was brought back for a two-week post-operative evaluation, displaying incomplete wound closure. (**G**) Nevertheless, tissue maturation was observed before implant placement at 3 months post-extraction. (**H**) Full-thickness flaps were reflected to display adequate bone volume. (**I**) The implant was then placed uneventfully.

The above case with partial loss of the e-PRF membrane led to the development of adding a standard Solid-PRF membrane as an additional external layer. Generally, Solid-PRF membranes are more robust than e-PRF with respect to their clinical handling. For these reasons, Solid-PRF was utilized in conjunction with an e-PRF membrane to minimize material loss. During its fabrication, while the e-PRF membrane is setting in the Bio-Heat tray (Bio-PRF, Jupiter, FL, USA), a Solid-PRF membrane can be placed over top of it, which allows them to set together as one dual-layered membrane (Figure 7). This technique involves placing the dual-layered PRF membrane in the extraction socket with the Solid-PRF facing towards the oral cavity. Since the Solid-PRF membrane will release its entire concentration of growth factors over 2 weeks, and the fact that it is more robust in nature, even if complete closure is not achieved during that timeframe, the extended-PRF layer will be present and fully intact to protect the graft particulate from escaping during socket preservation. A great example of this is demonstrated in Figure 8, in which the patient presented for a two-week post-operative evaluation, with the Solid-PRF membrane having completely resorbed, with the e-PRF membrane remaining to protect the graft material. One potential advantage of this technique is its perceived ability to speed soft tissue healing over the defect. Since Solid-PRF can release growth factors within 14 days instead of 4–6 months, it is evident that more growth factors are available earlier in the regeneration process, favoring faster soft tissue healing/closure. Alternatively, a Solid-PRF membrane can also be applied after placing e-PRF intra-orally, as highlighted in Figure 9.

While both above-mentioned iterations of utilizing e-PRF demonstrated excellent clinical outcomes, one of the primary disadvantages has been the added clinical time required to fabricate the e-PRF membrane, specifically having to wait extra time for the membrane to fully polymerize into a stable and fully clotted form. In total, 8 min are required for blood centrifugation to separate cell layers and produce both Solid- and Liquid-PRF. After the PPP layer is drawn, it must be placed in the Bio-Heat (Bio-PRF, Jupiter, FL, USA) for 10 min. After 2 min of cooling, the remaining Liquid-PRF buffy coat zone is then mixed with the albumin gel approximately 10 times to create the final Alb-PRF. Following placement in the e-PRF tray, it generally will take an extra 15 min for the membrane to fully clot/set within the desired shape. Therefore, fabrication of the e-PRF membrane may take up to 30–40 min, with the final step being the longest. In clinical practice, this may extend the surgical procedure as the extraction is often completed prior to membrane fabrication.

To overcome this limitation, it was hypothesized that the e-PRF clotting (final membrane polymerization) could be completed intra-orally. Two key breakthroughs were responsible for this development. The first was the fact that in orthopedic joint injections and in facial esthetics, the use of the extended-PRF (or Alb-PRF) had been utilized for several years as an injectable ‘Bio-Filler’ [28]. This technology is superior for knee injections, temporomandibular joint injections and has been utilized in facial esthetics as a replacement to common synthetic hyaluronic acid fillers such as Juvéderm and Restylane [28]. Therefore, the protocol to create the Bio-Filler, including its step-by-step procedure of fabrication, has been well established over a number of years in the medical community.

The second hypothesized advantage is that the clotting cascade of liquid fibrinogen and thrombin towards a fibrin clot is an enzymatic process. Enzymes function better at body temperature. Therefore, an attempt was made to perform the clotting and final step of e-PRF fabrication completed within the oral cavity. This led to iterations #3 and then #4 towards developing the ideal fabrication of e-PRF for use intra-orally for extraction site management.

### 3.3. Fabrication of e-PRF Intra-Orally in Gel Form, Also Known as the Bio-Filler Method

The advantage of fabricating e-PRF intra-orally is that it dramatically reduces the clinical time required to utilize this novel technology and is the least technique-sensitive. Following bone grafting of the socket, a cross suture is then applied over the “Sticky bone” and before application of the e-PRF in gel form (Bio-Filler). While it may not be necessary, the suture provides a matrix which may add stability to the Alb-PRF both prior to and after clotting. In the oral cavity, the e-PRF will form faster at body temperature than when setting chairside, as the conversion of fibrinogen plus thrombin into a fibrin clot is enzymatic and therefore functions better at body temperature. The patient can then immediately be sent home, reducing chair time. In these cases, patients are advised not to eat or drink for 2 h post-operatively to ensure adequate time for the membrane to fully stabilize.

Briefly, an 18 G needle is utilized to draw two 10 mL Liquid-PRF tubes of blood from the patient and centrifuged at 700× *g* for 8 min utilizing a horizontal centrifuge (Bio-PRF, Jupiter, FL, USA). After centrifugation, one syringe is utilized to draw 2 mL of the PPP layer from one blue tube and then placed in the Bio-Heat Device (Bio-PRF, Jupiter, FL, USA) for 10 min at a temperature of 75 degrees Celsius for serum albumin denaturation. This produces the albumin gel with a 4–6-month resorption time. A second syringe is then utilized to draw 0.5 mL of Liquid-PRF from the buffy coat zone and placed in the Bio-Cool (Bio-PRF, Jupiter, FL, USA) at a temperature of 8 degrees Celsius for 1–2 min before mixing. Both the Liquid-PRF buffy coat zone (0.5 mL) and the Albumin-PRF (2 mL) are then removed from the Bio-Cool device (Bio-PRF, Jupiter, FL, USA) and mixed slowly with a female–female luer lock until the gel is uniform (roughly 10 passes). The extended-PRF in Bio-Filler form is then ready for use.

**Figure 10:**
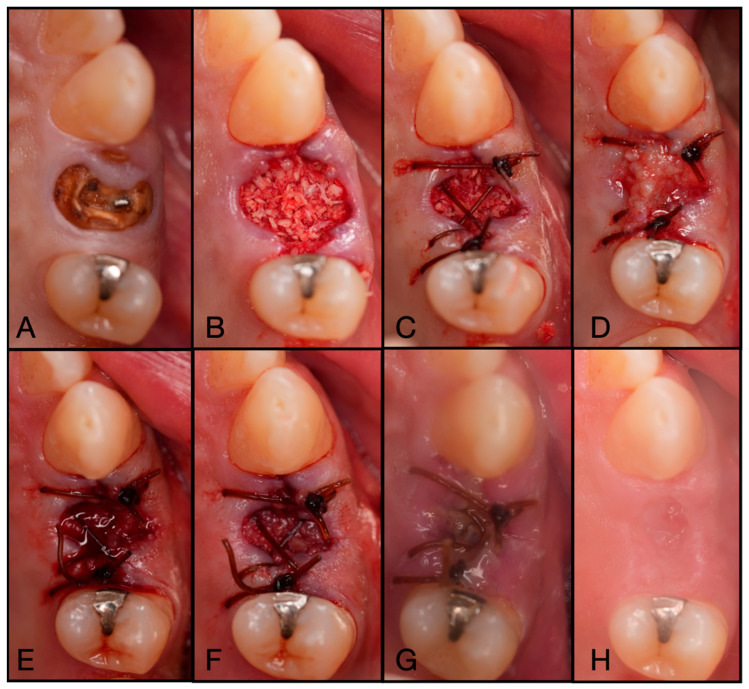
(**A**) Pre-operative photo of tooth #24 fractured to the gingival margin. (**B**) Following atraumatic extraction, the socket was degranulated with a spoon excavator and packed with “Sticky bone” created by mixing both Solid- and Liquid-PRF with particulate bone allograft. (**C**) A 4.0 chromic gut mattress suture before application of the Bio-filler membrane. (**D**) The soft tissue portion of the socket was filled with the Bio-filler membrane. The e-PRF membrane after (**E**) 30 min and (**F**) 60 min after application. (**G**) The patient was brought back 2 days post-operatively to verify that the e-PRF membrane was still present. (**H**) At 2 weeks post-operatively, complete wound closure is noted.

Figure 10 depicts the process of utilizing this novel evolution of the e-PRF membrane. This presents many advantages over previous iterations by both being (1) better adapted to the defect site with a more customized membrane shape and thickness, along with (2) saving roughly 15 min in chairside time owing to the final clotting process taking place within the oral cavity at body temperature instead of room temperature, allowing the procedure to be completed earlier.

### 3.4. Fabrication of the e-PRF Membrane Intra-Orally Under a Solid-PRF Membrane

**Figure 11:**
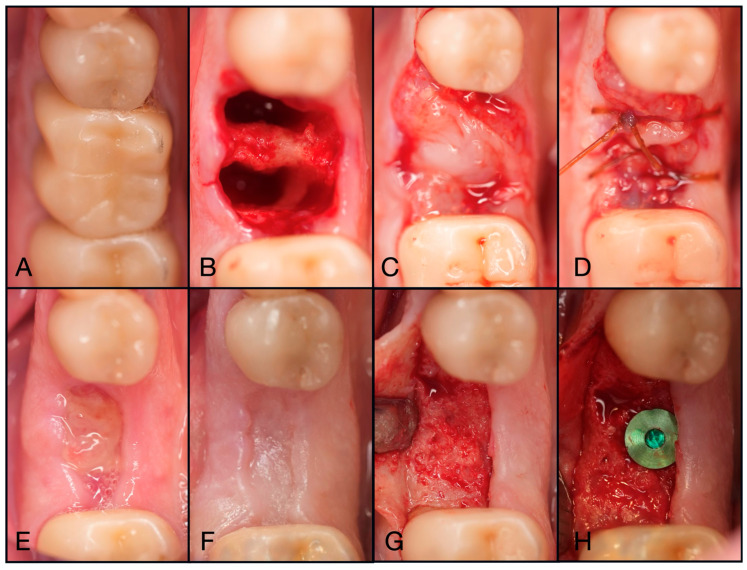
(**A**) Pre-operative clinical photograph of tooth #46, which presented with furcal decay and was deemed unrestorable. (**B**) After atraumatic extraction of tooth #46, the socket was degranulated with a spoon excavator and packed with “Sticky bone” created by mixing both Solid- and Liquid-PRF with particulate bone allograft. (**C**) A Solid-PRF membrane was then loosely placed over the “Sticky bone,” and the Bio-Filler was then injected underneath the Solid-PRF membrane utilizing an 18-gauge needle. (**D**) Then, 3-0 Chromic Sutures were utilized in a criss-cross fashion to secure the Solid–PRF and Bio-Filler in place. (**E**) The patient was brought back for a two-week post-operative evaluation, displaying excellent wound closure. (**F**) Tissue maturation was observed pre-operative to implant placement at 3 months post-extraction. (**G**) Full-thickness flaps were reflected, displaying adequate bone volume and quality. (**H**) The implant was placed uneventfully in adequate bone.

**Figure 12:**
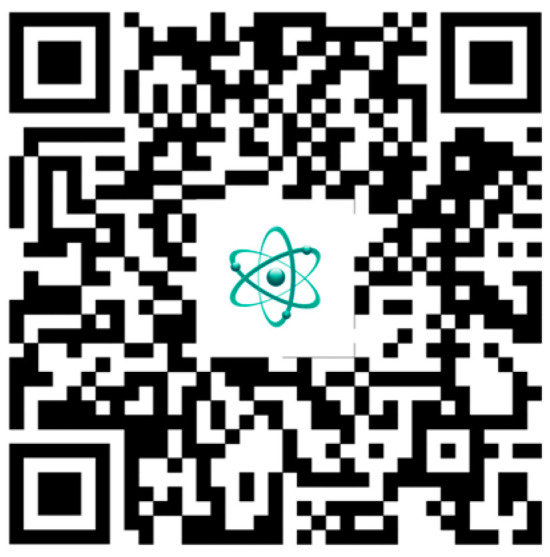
QR Code 3. Clinical demonstration of fabricating an e-PRF membrane intra-orally under a Solid-PRF membrane.

One of the concerns with iteration #3 has been related to patient compliance and the potential loss of the e-PRF within the first 2 h post-operatively. Similar to the development of iteration #2, an extra safety measure was utilized by placing a Solid-PRF membrane loosely over top of the Bio-Filler membrane, or by injecting the Bio-Filler (extended-PRF remaining in gel form) using an 18-gauge needle below/underneath the Solid-PRF membrane (Figure 11). Unlike the previous iteration, the suturing can be completed after the application of the Bio-Filler, providing additional stability (Figure 12, QR Code 3).

## 4. Discussion

The first article of this two-part series was designed to explain and showcase the iterations of the novel e-PRF membranes being utilized in clinical practice as a barrier during ridge preservation (Table 1). Over the past 3 years, a number of different techniques have been employed to fabricate e-PRF, with four different techniques demonstrated within this current study. The second article of this two-part series will evaluate and compare these various iterations with each other and to traditional collagen barrier membranes. While this paper was focused on the technique, observations of the success of these membranes are purely anecdotal, and a clinical trial is necessary for an accurate comparison. Two critical questions arise as follows: (1) Is there a compromise in soft tissue healing and success rates by having the final clotting step occur within the patient’s oral cavity? Also, (2) Is there any additional benefit to placing a Solid-PRF membrane overtop of the e-PRF membrane/Bio-Filler?

Note from the final clinical case presented above that by utilizing the Bio-Filler to create the final e-PRF membrane, the working time was reduced by 10–15 min by preventing the need for having the e-PRF membrane set in the Bio-Heat tray (Bio-PRF, Jupiter, FL, USA) for about 15 min to set fully. Once completed, the e-PRF membrane then needs to be properly shaped according to the defect, and during this process may become slightly damaged. By utilizing the Bio-Filler techniques (iterations 3 and 4), the liquid/gel-like properties favor better adaptation within the walls of the defect with a more even distribution. Additionally, this also allows for an easier ability to control the thickness of the final e-PRF membrane, whereas in the Bio-Heat tray (Bio-PRF, Jupiter, FL, USA), the membranes are typically thicker (1.5–2.5 mm), which may not always be convenient in certain cases, specifically in the anterior region.

Noteworthy, for these cases, patients are advised not to eat or drink for 2 h post-operatively to provide adequate time for the Bio-filler to fully set in the oral cavity. A second noteworthy clinical finding observed is that the ideal final membrane should be slightly apical to the gingival margin. This may allow for easier and faster soft tissue closure over the defect, as the membrane does not need to be resorbed and replaced for this to occur. A comparative study is necessary to compare the various techniques on soft tissue healing and soft tissue thickness at the time of implant placement.

One clinical feature that was observed over having performed many such cases was that, despite losing the e-PRF membrane in a few cases at early time points, in all cases, adequate bone formation was observed for implant placement (data found in part two of these articles). It may be worth noting that it is the authors’ belief that the “Sticky bone” consistency and density, which is inclusive of standard PRF protocols, may aid in providing cell exclusivity and/or keeping the graft material intact regardless of technique, potentially leading to positive new bone formation even despite membrane loss. This theory naturally requires further research and further validation, likely in an animal model, to fully investigate at the histological level various scenarios that may occur in clinical practice. Additionally, while the loss of the e-PRF membrane at the two-week post-operative evaluation in the first iteration of this technique led to the development of iteration #2, it is worth noting that early membrane degradation/exfoliation is a common complication of all membranes utilized in alveolar ridge preservation, including conventional membranes such as ePTFE and resorbable collagen [31,32]. Well-designed clinical trials are warranted to evaluate clinician-reported wound healing outcomes between conventional non-autologous membranes and the various techniques described in this paper.

Recently, our group has published a first case series utilizing the novel e-PRF for lateral window sinus closure [30]. In that study, the use of e-PRF membranes similar to iteration 1 was utilized [30]. While use of this technology continues to be proposed and developed, it would be of interest to investigate the Bio-Filler method for coverage of the lateral sinus window (among other procedures utilizing e-PRF) as this technique may also save the treating clinician chair time. Additionally, in a recent study by Barakat et al. [33], Alb-PRF was utilized as a standalone biomaterial for the sinus lift itself demonstrating an average of 5.07 ± 1.78 mm in vertical bone gain. Furthermore, a recent randomized clinical trial by Abdulhak et al. [29] compared the use of Alb-PRF to a connective tissue graft (CTG) on the modification of the gingival phenotype. In that study, twenty treatment sites were included in a split-mouth design involving 10 patients with a thin gingival phenotype in the mandibular anterior region. The results found that Alb-PRF application for modifying thin gingival phenotypes proved to be an effective therapeutic option, resulting in increased gingival thickness. It was also concluded that CTG demonstrated a greater enhancement in keratinized tissue width [29]. Despite these interesting and recent findings, it would be of significance to compare the outcomes of the novel Alb-PRF/e-PRF to collagen membranes for recession coverage. Furthermore, the various techniques proposed in this article, such as utilizing the Bio-Filler method, may also be proposed as a simpler modality for recession coverage using Alb-PRF, with further investigation needed to determine its effectiveness in vivo.

The first article of this two-part series was designed as a technical narrative review to demonstrate four unique iterations of the novel e-PRF membranes being utilized in clinical practice as a barrier during ridge preservation. However, a significant limitation of the current study is the lack of comparison among the different techniques and traditional barrier membranes. In the second part of this series, a randomized clinical trial is conducted to evaluate the e-PRF variations in comparison to traditional collagen membranes, with outcome variables including horizontal and vertical ridge dimensions, soft tissue thickness, clinician-reported healing outcomes, and the time required for membrane fabrication.

## 5. Conclusions

In this first article of this two-part series, the various techniques for how to utilize e-PRF in clinical practice for ridge preservation were depicted with step-by-step photos. A logical flow was presented for how the technique has evolved by assessing practical challenges, and four iterations for how the technology may be utilized in clinical practice are presented with standardization of the fabrication process. In part two of this series, a comparative study will provide further quantitative evaluation and comparison between the four techniques and traditional collagen membranes. Today, PRF is available in both solid and liquid forms; however, this novel formulation of heated-PRF to create Alb-PRF membranes has presented many new opportunities across many fields of medicine, including dentistry. This novel technology was given the working name ‘extended-PRF’ or e-PRF, with many clinical indications being proposed for further study as a substitute for collagen barrier membranes.

## Figures and Tables

**Table 1:** Summary table providing an overview of all four ePRF iterations in ridge preservation.

Technique	Centrifugation	Tubes	Bio-Heat (75 °C)/ Bio-Cool (8 °C)	Extraoral Setting	Dual Layer w/Solid-PRF	Suturing
Membrane	700 g for 8 min	2 Blue tubes	10 min/optional	Yes (~15 min)	No	After application
Membrane w/Solid	700 g for 8 min	2 Blue tubes, 1 red tube	10 min/optional	Yes (~15 min)	Yes	After application
Bio-Filler	700 g for 8 min	2 Blue tubes	10 min/2 min	No	No	Before application
Bio-Filler w/Solid	700 g for 8 min	2 Blue tubes, 1 red tube	10 min/2 min	No	Yes	After application

## Data Availability

The original contributions presented in this study are included in the article. Further inquiries can be directed to the corresponding author.
